# Harnessing myostatin pleiotropy for multitrait improvement via precision gene editing

**DOI:** 10.3389/fgeed.2026.1749445

**Published:** 2026-03-12

**Authors:** Yajun Chen, Ruiyao Yang, Yucai Yang, Qianguang Wang, Kai Yang, Man Xu

**Affiliations:** 1 Changde Vocational and Technical College, Changde, Hunan, China; 2 Yunnan Haorui Agricultural Development Co., Ltd., Kunming, Yunnan, China; 3 Yunnan Academy of Grassland and Animal Science, Kunming, China

**Keywords:** CRISPR/Cas9, gene editing, MSTN, pleiotropy, sustainable breeding

## Abstract

The pursuit of sustainable livestock farming to meet the rising global protein demand has positioned myostatin (MSTN) gene editing as a key technology. However, the field’s focus on the remarkable double-muscle phenotype has often overshadowed a systematic examination of its concomitant effects. The present review aims to bridge this gap by moving beyond a singular focus on productivity. First, the pleiotropic effects of MSTN gene editing on growth performance, carcass quality, and meat quality in cattle, swine, sheep, poultry, and aquatic species were comprehensively evaluated. Next, the cascading biological effects of MSTN editing on metabolic homeostasis, reproductive performance, and animal health and welfare werAAe analyzed in depth. Finally, the inherent limitations and ethical issues of current editing techniques were critically discussed, and future sustainable breeding programs aimed at balanced multitrait regulation were prospectively proposed. Ultimately, this review affirms that MSTN editing has a multiplicative effect on trait alterations; however, there is also a series of associated health challenges, which demonstrates that the technology’s impact is systemic, generating a spectrum of trade-offs that are often species specific. Its responsible application therefore hinges on multitrait balancing strategies to simultaneously secure productivity and sustainability in animal agriculture.

## 1 Introduction

Global population growth and increasing consumption levels have led to increased demands for animal-derived food, creating an urgent need to promote the transformation of animal husbandry toward high-yield, high-quality, and sustainable production (Wang et al., 2025). Traditional breeding techniques have made important contributions to the genetic improvement of livestock; however, long breeding cycles and slow genetic progress have become increasingly challenging. In addition, the gaseous reactive nitrogen emissions generated during breeding exert pressure on the environment, emphasizing the need for more efficient and environmentally friendly animal breeding strategies (Yu et al., 2025). In this context, rapid advances in molecular breeding technologies, particularly gene editing, offer new technical pathways for the genetic improvement of livestock and poultry. Among them, the myostatin (MSTN) gene, also known as growth differentiation factor 8 (GDF-8), is a key negative regulatory factor in skeletal muscle growth and has emerged as a gene of considerable potential in livestock breeding (Kalds et al., 2023).

MSTN is a member of the TGF-β superfamily. Through gene knockout experiments, it was first confirmed in 1997 that MSTN inhibits the proliferation and differentiation of myoblasts through autocrine and paracrine mechanisms, precisely regulating muscle mass (McPherron et al., 1997). A loss-of-function mutation of this gene can reduce its inhibitory effect on muscle growth, leading to the typical “double-muscle” phenotype in animals. This phenomenon has been validated in natural mutant breeds such as Belgian blue cattle and Piedmontese cattle (Lee EJ. et al., 2021). The successful replication of this phenotype via targeted gene editing in various livestock species has further solidified the status of MSTN as a high-priority target for increasing meat yield (Lee et al., 2024; Hickford et al., 2010).

However, the singular focus on double-muscle traits has led to neglect of the comprehensive effects brought about by MSTN gene editing. Studies have shown that MSTN contributes to the regulation of muscle growth and participates in the aging process. Findings have also indicated that MSTN contributes to the regulation of muscle aging by upregulating the expression of the fibromodulin (FMOD) gene. The FMOD protein, in turn, affects MSTN transcription or activity via a negative feedback mechanism, resulting in the formation of a negative feedback loop that regulates muscle aging (Grochowska et al., 2019). The scientific discourse on MSTN has been dominated by its double-muscle effects, but its function as a molecular integrator of systemic metabolic homeostasis is poorly understood. This knowledge gap has caused an insufficient assessment of the concomitant physiological compromises—such as metabolic shifts, reproductive challenges, and health issues—that accompany enhanced musculature across different species (Hickford et al., 2010; Ayuti et al., 2024). Therefore, advancing the field toward responsible application of MSTN editing necessitates a holistic and comparative understanding of its pleiotropic networks. The core objective of this review is to provide this systemic perspective. We systematically evaluate the pleiotropic effects of MSTN editing across major livestock species, analyze the underlying biological trade-offs, and discuss pathways toward sustainable breeding through balanced multitrait regulation.

## 2 Biological characteristics of the MSTN gene

### 2.1 Gene structure

The MSTN gene is highly conserved in animals. Its genomic structure exhibits the typical structural characteristics of TGF-β superfamily genes, which contain three exons and two introns. The encoded precursor protein undergoes two protease hydrolysis processes to generate the biologically active C-terminal mature peptide (Grobet et al., 1997). This peptide forms a homodimer through the “Cys-Knot” structure, which is formed by nine conserved cysteine residues. This structure is essential for maintaining the spatial conformation and biological functions of the peptide (Kambadur et al., 1997).

Although the MSTN gene sequence is highly conserved, it still differs to some extent among different species (Gong et al., 2009). For example, the MSTN gene in mice encodes 376 amino acids, whereas in other mammals, it encodes only 375 amino acid residues (Hill et al., 2010). Birds have five different splice variants (MSTN-A to MSTN-E), of which MSTN-A has complete biological activity, while MSTN-B has a certain antagonistic function because it lacks part of the propeptide region (McPherron et al., 1997; Maeta et al., 2022). Naturally occurring loss-of-function mutations further confirm the gene’s functional conservation. The frameshift mutation caused by the 11-bp deletion in Belgian blue cows and the G→A missense mutation in Piedmontese cows could cause premature termination of translation and eventually produce a “double-muscle” phenotype (Lin et al., 2002; Kerr et al., 2005). Therefore, although the homology of the coding regions is high, the functions of transcription regulatory mechanisms, expression profiles, and splice variants of MSTN genes in different species may significantly differ. These variations provide a molecular basis for cross-species comparative studies and the optimization of targeted breeding strategies.

### 2.2 Expression and regulatory mechanisms

The expression of the MSTN gene is clearly specific to each tissue and developmental stage, with the highest expression occurring in animal skeletal muscle (Grobet et al., 1997). This gene is expressed mainly in somite and muscle progenitor cells during the embryonic stage and regulates the number of muscle fibers; after birth, it is expressed mainly in muscle fibers to regulate total muscle mass (Philip et al., 2005). In addition to skeletal muscle, MSTN is expressed at lower levels in the myocardium, adipose tissue, liver, and kidney (Li et al., 2021). These findings indicate that MSTN may be involved in the regulation of multiorgan metabolism. This gene is more widely expressed in fish and can be detected in gill, intestine, and gonad tissues (Clop et al., 2006), reflecting species-specific differences in adaptive expression.

MSTN expression is finely regulated at multiple levels. At the transcription level, promoter activity is regulated by key myogenic transcription factors such as MyoD and MEF2 (Zhang et al., 2018). At the posttranscription level, a variety of microRNAs (such as miR-1, miR-206, and miR-27b) can inhibit the translation of MSTN mRNA through targeted binding to the 3′-untranslated region (3′-UTR) (Morissette et al., 2009; Rodgers and Garikipati, 2008). A G→A mutation in the 3′-UTR of the MSTN gene in Texel sheep introduces a new miR-1/206 binding site, resulting in decreased MSTN expression and a muscle hypertrophy phenotype (Rodgers and Garikipati, 2008). This finding indicates that economic traits can be improved through the regulation of miRNA expression. In addition, MSTN upregulates the expression of miR-27a/b through Smad3, and conversely, miR-27a/b can target and inhibit MSTN, thereby introducing a new type of miRNA-mediated self-regulatory negative feedback loop in the myogenesis process (McFarlane et al., 2014). Other studies have shown that environmental factors such as heat stress and high stocking density can also significantly upregulate MSTN expression to further inhibit muscle growth (Li et al., 2019). These findings indicate that optimized feeding management is highly important for the realization of genetic potential.

### 2.3 Signaling pathways and molecular mechanisms

At the molecular level, MSTN signaling converges on a multilevel regulatory system centered on the Smad pathway, including extensive cross-talk with other critical networks that collectively determine its pleiotropic outcomes.

**FIGURE 1:**
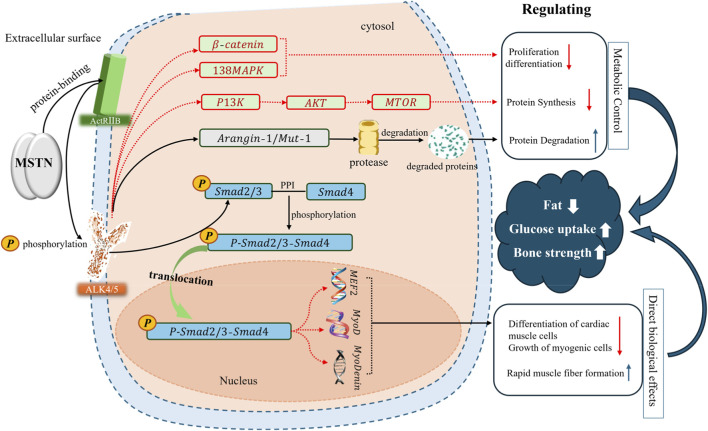
Schematic illustration of the intracellular signaling pathways regulated by myostatin (MSTN). Legend: MSTN primarily inhibits key transcription factors, such as MyoD and MEF2, through the classic Smad pathway (indicated by the black arrow), which activates receptors and facilitates the nuclear translocation of the Smad2/3–Smad4 complex, thereby restricting muscle growth. Simultaneously, MSTN inhibits anabolic pathways such as PI3K/Akt/mTOR, as shown by the red dotted lines, and activates the p38/JNK MAPK pathway, jointly regulating protein synthesis and degradation.

MSTN functions mainly through the typical TGF-β/BMP signaling pathway (Figure 1) (Morissette et al., 2009). By binding to the type II receptor (ACVRIIB) on the cell membrane, the mature MSTN dimer recruits and phosphorylates the type I receptor (ALK4 or ALK5) to activate downstream Smad2/Smad3 signaling proteins. The activated Smads then form a complex with Smad4 and translocate into the nucleus (Goodman et al., 2013). In the nucleus, this complex acts as a transcriptional regulator to directly inhibit the expression of key myogenic regulatory factors such as MyoD and myogenin, thereby inhibiting the proliferation and differentiation of myoblasts (Hennebry et al., 2009).

In addition to the Smad pathway, MSTN contributes to the inhibition of muscle growth through multiple mechanisms. Its signaling activity can intersect with the P13K/Akt/mTOR pathway, thereby inhibiting protein synthesis, increasing the expression of E3 ubiquitin ligases (such as Atrigin-1 and MuRF-1) to promote protein degradation, and jointly restricting muscle hypertrophy through degradation and synthesis (Goodman et al., 2013; Hennebry et al., 2009). MSTN also activates the p38 MAPK and JNK pathways through TAK1–MKK6 signaling and inhibits myoblast proliferation independent of the Smad pathway (Carnac et al., 2007). Additionally, the Arangin-1/Mut-1 pathway has been implicated in MSTN signaling, further expanding the regulatory network governing muscle homeostasis. In addition, MSTN can regulate muscle fiber type conversion. By inhibiting transcription factors such as MEF2 and MyoD, MSTN can promote the conversion of muscle fibers from oxidative to glycolytic, thereby affecting muscle metabolism (Hennebry et al., 2009; Joulia et al., 2003). Together, these pathways constitute a complex multilevel regulatory system to finely control muscle homeostasis.

These pathways collectively form a complex, multilevel regulatory system that finely controls muscle homeostasis. It is worth noting that MSTN’s signaling specificity is not absolute. MSTN shares up to 90% sequence homology with the mature peptide sequence of its homolog GDF11, and the two of them share the ActRIIB and ALK4/5 receptors as well as the downstream Smad2/3 signaling molecules (Chen et al., 2021). However, GDF11 is more focused on regulating tissue aging and embryonic development (Chen et al., 2021; Walker et al., 2016). This receptor sharing and functional differentiation implies that interventions targeting MSTN may inadvertently disrupt GDF11 signaling, thereby producing effects far beyond the muscular system.

### 2.4 Pleiotropic regulatory functions

The pleiotropic effects induced by MSTN editing stem from its function as a core node in the GDF11/BMP signaling network. Therefore, its loss-of-function phenotype should be understood as triggering a systemic remodeling of the GDF11/BMP signaling network rather than a simple lifting of muscle growth inhibition. This network-level reprogramming coordinates multiple mechanisms to influence various systems in the body, including metabolism, skeletal structure, and reproduction.

In terms of metabolic homeostasis, MSTN’s inhibitory effect on muscle anabolism via the Smad2/3 pathway also extends to systemic metabolic regulation. Its functional deficiency alleviates the inhibition of insulin/AKT signaling, thereby increasing glucose uptake and improving insulin sensitivity (Liu et al., 2018; Ahmad et al., 2018). More importantly, the weakened MSTN signal alters the competition for shared effectors (such as ActRIIB) and intracellular signaling components (such as Smad4), potentially favoring the activation of other metabolic regulatory signals within the TGF-β superfamily network (Sartori et al., 2013). This network-level rebalancing, achieved in part by downregulating key lipogenic factors such as PPARγ, collectively results in a significant reduction in fat deposition (Clop et al., 2006).

In terms of bone metabolism, the observed increases in bone density and strength are a direct result of the compensatory activation of the GDF11/BMP pathway (Bialek et al., 2014). When MSTN signaling is reduced, the subsequent upregulation of GDF11 expression and release of BMP signaling capacity jointly increase the activity of the pro-osteogenic Smad1/5/8 pathway (Sartori et al., 2013; Suh et al., 2020). This mechanistic linkage directly couples muscle hypertrophy phenotypes with skeletal adaptations and explains species-specific effects, such as increased bone density in quails and skeletal developmental abnormalities in mammals, by accounting for variations in the baseline state and output of this compensatory network across different species.

This network influence also extends to critical systems such as the reproductive and cardiovascular systems. In the cardiovascular system, modulating cardiac remodeling by suppressing MSTN-induced network perturbations provides protection against pathological hypertrophy (Yang et al., 2023). In terms of reproduction, systemic metabolic shifts and altered endocrine environments triggered by the rebalancing of the MSTN–GDF11–BMP network may indirectly influence reproductive processes. Furthermore, the direct expression in the gonads suggests local effects, potentially influencing follicular development and steroid production in females as well as Leydig cell function in males (Wang S. et al., 2022; Gonzalez-Ponce et al., 2022). It is worth noting that gene-edited animals face reproductive challenges, such as declining litter sizes in pigs and delayed onset of egg-laying in poultry (Tivesten et al., 2002), which may stem from the signal’s direct action in the gonads or may represent the body’s redistribution of resources to prioritize metabolism and skeletal growth.

In summary, the MSTN–GDF11–BMP signaling network functions as an integrated regulatory module. Editing MSTN triggers a series of compensatory adjustments within this module, radiating to coordinated changes in the muscular, metabolic, skeletal, and reproductive systems. This process elucidates how diverse polygenic effects represent interconnected manifestations of the same systemic regulatory disorder across different physiological levels.

## 3 MSTN gene-editing technology

The elucidation of the multilevel biological mechanisms involving MSTN points to a core challenge: how can this system be precisely regulated to achieve predictable breeding phenotypes and manage its pleiotropy? This challenge has directly driven the iterative development of gene-editing tools. The evolution from zinc finger nucleases (ZFNs) and transcription activator-like effector nuclease (TALENs) to CRISPR/Cas9 and its derivative systems represents a continuous process of optimizing precision, efficiency, and controllability for this specific target, and it also charts the path from fundamental understanding to agricultural application.

### 3.1 Evolution of editing tools

The iterative development of gene-editing technology has provided a powerful tool for functional analysis and breeding applications involving the MSTN gene. Early techniques, such as ZFN and TALEN gene editing, have enabled targeted gene knockout. For example, using ZFN technology, Qian et al. (2015) successfully induced a double-muscle phenotype in Meishan pigs. However, these techniques have limitations such as complex designs and high costs. In recent years, the CRISPR/Cas9 system has become the mainstream technology for MSTN gene editing because of its simple design, high efficiency, and low cost (Cong et al., 2013). This system uses Cas9 nuclease to generate DNA double-strand breaks at the target site and uses the cell’s own nonhomologous end joining repair mechanism to introduce insertion/deletion mutations, thereby achieving gene knockout. This technique has been successfully applied in cattle (Gim et al., 2022a), sheep (Zhou et al., 2022), pigs (Cong et al., 2013), poultry (Kim et al., 2020), and aquatic animals (Khalil et al., 2017). This evolution from ZFNs and TALENs to the prevailing CRISPR/Cas9 platform has firmly established gene editing as the foremost method for functional studies of MSTN and the induction of the double-muscle phenotype across species.

### 3.2 CRISPR/Cas9 system optimization strategy for the MSTN gene

To improve the efficiency and safety of CRISPR/Cas9 in breeding, various optimization strategies have been implemented. For target selection, the conserved regions in the front exons of the MSTN gene that encode key functional domains were screened through bioinformatics analysis to maximize the effect of loss-of-function mutations (Kong et al., 2025). To improve efficiency, researchers have significantly increased the success rate of gene editing by optimizing the secondary structure of guide RNAs (gRNAs), using high-fidelity Cas9 variants, and improving embryonic microinjection (Gim et al., 2022a). In terms of controlling off-target effects, the dual-nickase system (D10A-Cas9), the use of high-fidelity Cas9 variants, and the analysis of whole-genome off-target effects (such as GUIDE-seq) greatly increase the safety of editing (Zhou et al., 2022). However, the editing strategy needs to take species differences into account. For example, owing to the special structure and reproductive physiology of zygotes, the efficiency of conventional embryo injection in poultry is low. Thus, special methods, such as adenoviral vectors or primordial germ cell (PGC) transplantation, are often used for gene editing (Kim et al., 2020; Lee et al., 2022).

### 3.3 Next-generation editing tools for the precise regulation of MSTN function

Currently, CRISPR/Cas9-mediated MSTN gene knockout can effectively increase muscle yield; however, random mutations are difficult to control, and complete loss of function is often accompanied by side effects such as reproductive disorders and skeletal abnormalities (Zhou et al., 2022; Khalil et al., 2017). Next-generation gene editing tools, particularly base editors (BEs) and pre-editors (PEs), offer promising approaches for overcoming these bottlenecks (see Table 1). The primary advantage of these systems lies in their shift from “complete knockout” to “precise regulation” (Anzalone et al., 2019). For example, a base editor can effectively simulate natural missense mutations, such as the GDF8 point mutation in Piedmontese cattle, rather than introducing random breakage, which is expected to reduce the negative effect caused by the complete absence of the protein while maintaining muscle growth (Meloux et al., 2019). Pre-editors allow more complex “customized” editing, such as introducing precise and subtle mutations in the regulatory region of the MSTN gene rather than completely eliminating its expression. This approach offers unprecedented potential for balancing muscle growth and animal health or reproductive performance (Anzalone et al., 2019).

**TABLE 1:** Comparison of the characteristics of major gene-editing technologies.

Technology platform	Major advantage	Main limitations	Editing efficiency	Relative cost	Phase of application to MSTN
ZFN	Customized gene editing	Off-target effects, high toxicity	Medium (Pagant et al., 2021)	Extremely high (Wang et al., 2022b)	Early proof-of-concept studies (e.g., in pigs) (Qian et al., 2015)
TALEN	More flexible with higher specificity	Cloning and delivery challenges	Medium to high (Mahata and Biswas, 2017)	High (Wang et al., 2022b)	Early proof-of-concept studies (e.g., in rabbits) (Lv et al., 2016)
CRISPR/Cas9	Simple design, capacity for re-editing (Cong et al., 2013)	Risk of off-target effects and PAM sequence restrictions	High (Alariqi et al., 2025)	Low (Wang et al., 2022b)	Mainstream technology, widely used in many species (e.g., cattle (Gim et al., 2022a), sheep (Zhou et al., 2022), and chickens (Kim et al., 2020))
Base editor (BE)	Low risk of off-target effects, high accuracy (Anzalone et al., 2019)	Limited types of editing	Medium (Tachida et al., 2025)	Medium	Frontier exploration stage. Reversible pathogenic mutation (Liang et al., 2023)
Pilot editor (PE)	Extremely high accuracy (Anzalone et al., 2019)	Complex system, low editing efficiency	Medium to low (Wei et al., 2025)	High	Frontier exploration stage. Partial restoration of vision in mice (An et al., 2024)

### 3.4 Application of MSTN gene editing in different species

**FIGURE 2:**
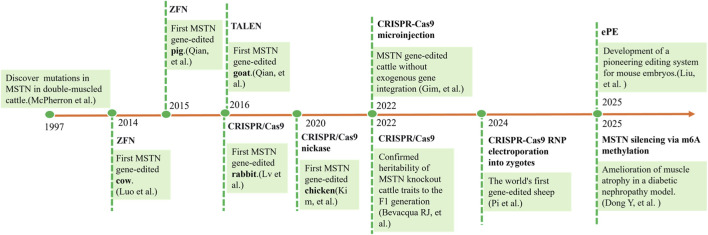
Timeline of MSTN gene-editing developments. Note: From the discovery of target points in 1997 to the validation of technical feasibility in 2014, design breeding was initiated. Through cross-species validation from 2015 to 2020, the core role of MSTN was confirmed, ultimately advancing to the current phase of industrial deepening since 2022, focusing on multitrait balance.

Since the first successful editing of the goat MSTN gene and the generation of knockout embryos using the CRISPR/Cas9 system combined with somatic cell nuclear transplantation by Ni et al. (2014), MSTN gene-editing technology has achieved a series of breakthroughs in livestock breeding and related fields (Figure 2). However, it has not yet been widely implemented in large-scale production. Subsequent studies have achieved efficient MSTN gene editing in multiple species through the optimization of various delivery systems: Crispo et al. (2015) generated MSTN-knockout sheep by microinjecting Cas9 mRNA and sgRNA into the cytoplasm of fertilized eggs, confirming the effectiveness of this technology in ruminants; Wang et al. (2015) developed MSTN-knockout pigs via somatic cell nuclear transplantation, demonstrating that CRISPR/Cas9 can efficiently induce MSTN gene mutations in pigs; Gim et al. (2023) demonstrated that the RNP electroporation method offers the advantage of improved efficiency in bovine embryo editing. The simultaneous knockout of MSTN and FGF5 genes in sheep through multigene collaborative editing marked a significant leap from single-trait improvement to comprehensive trait selection (Chen et al., 2024).

MSTN gene editing has achieved significant improvements in muscle development across various species. In livestock, Gim et al. (2022a) reported that MSTN-edited cattle exhibited an 8%–15% increase in carcass weight and a 15%–20% increase in loin-eye area. Sheep generated using an optimized Cas9/sgRNA delivery system showed an 8%–12% increase in body weight (Zhou et al., 2022). In pigs, MSTN editing not only increased muscle depth and reduced backfat thickness (Qian et al., 2015) but also improved glucose metabolism and insulin sensitivity (Li et al., 2020a). Regarding poultry, Kim et al. (2020) successfully knocked out the MSTN gene in chickens using the D10A–Cas9 nickase system, leading to a specific increase in muscle mass in the legs and wings. Studies in quail also revealed muscle hyperplasia and improved feed conversion efficiency (Lee et al., 2020). Regarding aquatic species, MSTN-edited catfish exhibited enhanced muscle growth (Khalil et al., 2017), while Liu et al. (2020) elucidated at the cellular level that MSTN editing influences cell proliferation via suppression of the mTOR signaling pathway. However, editing also has pleiotropic effects; for example, bone density increases before sexual maturity (Lee et al., 2022), resulting in negative effects on reproductive performance, such as reduced egg counts and delayed onset of laying (Lee et al., 2021b).

As MSTN gene editing technology matures, the research focus has shifted from merely increasing efficiency to improving biosafety and expanding the scope of application. The development of exogenous-DNA-free MSTN knockout cattle by Gim et al., along with the confirmation of stable heritability of the edited traits, has provided critical evidence for the safe application of gene-edited livestock (Gim et al., 2022a; Gim et al., 2022b). Concurrently, research frontiers are expanding into the medical field. Dong et al. (2025) engineered exosomes to silence MSTN expression by modifying m6A methylation, and these exosomes demonstrated therapeutic potential in a diabetic nephropathy model. This is a strategic transition of MSTN modulation from agricultural breeding to disease therapy. Collectively, these advances are driving the translation of MSTN gene editing from basic research toward practical application.

## 4 Pleiotropic effects of MSTN gene editing on production traits

When these cutting-edge editing tools are successfully applied to animal genomes, how do the final output “products”, that is, the growth, meat production, and health traits of MSTN gene-edited individuals, perform? These are the core elements for evaluating the breeding value of this technology and the key focus areas for future research.

### 4.1 Improvement in growth performance

**FIGURE 3:**
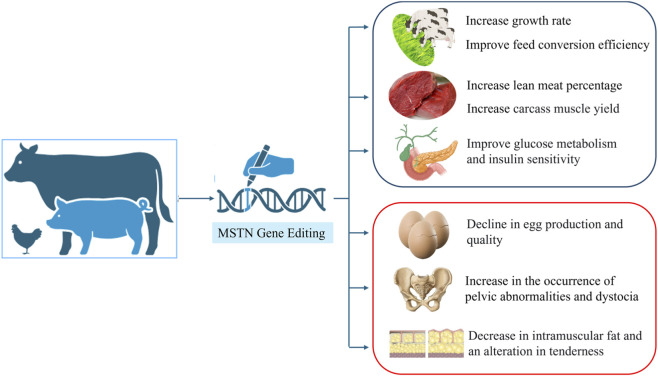
Pleiotropic effects of MSTN gene editing on production traits.

The loss of MSTN function generally leads to significant improvements in the animal growth rate and feed conversion efficiency, among other trait modifications (Figure 3). In mammals such as cattle and sheep, edited individuals usually show a continuous gain advantage during the entire growth period (Gim et al., 2022a; Zhou et al., 2022). In poultry, such as quails, the growth-promoting effect is greatest in the middle and late growth stages (Lee et al., 2020). This difference in the growth pattern of these species may be related to their respective growth and development patterns. The underlying mechanism is related mainly to energy metabolism. After the inhibition of muscle growth is removed, protein anabolism is enhanced, and the body allocates more energy to muscle tissue rather than to fat deposition, thereby improving overall feed utilization efficiency (Li R. et al., 2020; Zhao et al., 2022). Therefore, the growth-promoting effect of MSTN editing not only depends on its “growth switch” but is also subject to the species’ inherent growth curve and the fine-tuned regulation of metabolic timing.

### 4.2 Effects on carcass quality and meat quality

In terms of carcass quality, the most significant changes observed in MSTN-edited animals are a substantial increase in muscle yield and a decrease in fat deposition. Notably, there are species and sex differences in the location and extent of reduced fat deposition. For example, abdominal and leg fat were significantly reduced in quails (Lee et al., 2020). However, a significant reduction in abdominal fat was observed only in males (Kim et al., 2020). This phenomenon cannot be simply attributed to the passive change in energy distribution but is the result of MSTN directly regulating lipid metabolism pathways. Studies have shown that MSTN directly inhibits the process of lipogenesis by inhibiting the expression of the key adipogenic transcription factor PPARγ (Deng et al., 2017).

The effects on meat quality are more complex. A common trend is a decrease in intramuscular fat content, which may affect the flavor and juiciness of meat (Zhou et al., 2022; Kim et al., 2023). Changes in meat tenderness vary across species and parts, and the mechanisms underlying these changes may be related to changes in muscle fiber type composition and changes in calpain activity associated with postmortem muscle tenderization. MSTN editing may affect the glycolytic potential in muscle, leading to a change in the rate of decrease in the pH value of postmortem muscle, thereby affecting the color and water-holding capacity of the meat (Kim et al., 2023).

### 4.3 Effects on animal health

MSTN gene editing has demonstrated significant potential in enhancing meat production performance in livestock and poultry, yet it also impacts animal health to varying degrees. These effects can be systematically categorized into three major domains, namely, the skeletal system, reproductive health, and metabolic function, and exhibit both cross-species commonalities and species-specific characteristics (Gim et al., 2022a; Lv et al., 2016).

In the skeletal system, loss of MSTN function leads to significant muscle hypertrophy, which places a persistent burden on the bones and often results in structural defects. In cattle, this manifests as skeletal dysplasia and increased rates of dystocia (Gim et al., 2022a); in pig models, multiple developmental defects have been reported, including skeletal malformations (Qian et al., 2015); and abnormalities such as pelvic bone tumors have been observed in rabbits (Lv et al., 2016). Recent studies have shown that sheep engineered with dual gene editing of MSTN and FGF5 via CRISPR/Cas9 exhibit significantly enhanced muscle fiber proliferation. However, the long-term impact on skeletal load-bearing capacity remains to be evaluated (Chen et al., 2024). Notably, MSTN gene-edited quails have exhibited increased bone density (Lee et al., 2022), which suggests that the role of MSTN in bone metabolism has some degree of species specificity.

Second, impaired reproductive health represents another cross-species challenge. MSTN is widely expressed in female reproductive organs and participates in regulating key processes such as follicular development (Ongaro et al., 2025). In quails, this directly leads to reduced egg production, delayed onset of egg laying, and compromised eggshell quality (Kim et al., 2020; Kim et al., 2023; Lee et al., 2021c). Similarly, the reproductive performance of chickens faces potential risks (Gim et al., 2022a; Hai et al., 2023). In cattle, studies have reported upregulation of sperm-motility-related proteins (Zhao et al., 2022); however, the long-term effects on overall reproductive fitness remain unclear. In sheep, the long-term reproductive performance still requires comprehensive evaluation (Zhou et al., 2022).

MSTN editing profoundly restructures the body’s metabolic functions, yielding complex outcomes with both beneficial and detrimental aspects. In pigs, MSTN editing may improve glucose metabolism and insulin sensitivity (Cong et al., 2013). Recent studies in sheep have shown that dual gene editing of MSTN and FGF5 significantly remodels the metabolic profile of muscle satellite cells, thereby suppressing the tricarboxylic acid cycle and promoting the pentose phosphate pathway (Chen et al., 2023). This profound metabolic impact not only increases muscle yield but also influences the animal’s overall energy homeostasis. Additionally, MSTN gene editing improves the gut microbiota of sheep, yielding additional metabolic benefits (Kim et al., 2023; Lee et al., 2021d; Du et al., 2022), but its long-term physiological significance remains to be explored. Therefore, for both economic traits and health in animal production, MSTN editing should undergo a comprehensive and systematic evaluation before further advancement, and the potential of polygenic editing should be recognized (Chen et al., 2023).

### 4.4 Mechanistic analysis of species-specific effects and implications for breeding

The species differences presented in Table 2 stem from fundamental variations in growth and development patterns, reproductive system types, and energy allocation strategies among different species. At the developmental level, the sustained muscle growth pattern throughout the growth period in mammals (such as cattle and sheep) confers a continuous weight gain advantage after genetic editing. In contrast, the concentrated developmental characteristics of poultry (such as quail) result in a pronounced effect during the middle to late growth stages (Crispo et al., 2015). At the level of reproductive health, the differences between viviparity and oviparity lead to distinctly different challenges. Specifically, viviparous mammals (such as cattle and pigs) primarily face maternal–fetal conflicts, manifested as dystocia or reduced litter sizes (Tivesten et al., 2002; Gim et al., 2022a); the core conflict in oviparous birds lies in resource allocation, specifically, the trade-off between muscle growth and egg/shell formation, which leads to reduced egg-laying performance (Lee et al., 2021c; Hai et al., 2023; Chen et al., 2023). At the metabolic level, interspecies differences in active lipid metabolism pathways (e.g., in pigs) or unique “gut–muscle axis” regulation (e.g., in sheep) determine the extent of fat reduction and the magnitude of potential metabolic benefit (Li R. et al., 2020; Zhao et al., 2022; Hai et al., 2023). Therefore, the application of MSTN editing must adhere to the principle of “species-specific customization”: the core breeding strategy involves balancing muscle growth with reproductive health in mammals, coordinating muscle growth with reproductive resource allocation in poultry, and weighing growth advantages against early developmental vitality in aquatic animals. A deep understanding of these intrinsic biological principles is key to transcending mere descriptive analysis and achieving cross-species optimization of precision breeding.

**TABLE 2:** Pleiotropic effects of MSTN gene editing in different animal models.

Species	Muscle growth	Fat reduction	Health effects	References
Cattle	15%–20%	15%–25%	Increased dystocia; elevated sperm motility proteins	Hai et al. (2023), Zhao et al. (2022)
Pigs	10%–15%	20%–50%	Reduced litter size; improved metabolism	Qian et al. (2015), Li et al. (2020), Choe et al. (2022)
Sheep	10%–30%	\	Enhanced gut microbiota	Zhou et al. (2022), Du et al. (2022)
Rabbits	30%–40%	\	Abnormal pelvic development, bone tumors	Lv et al. (2016)
Fish	Approximately 40%	10%–20%	High fertilized egg mortality	Khalil et al. (2017), Liu et al. (2020)
Poultry	15%–25%	Approximately 15%	Decreased egg production, delayed onset of egg laying, reduced eggshell quality	Lee et al. (2022), Lee et al. (2020), Lee et al. (2021c)

## 5 Biological mechanisms underlying the pleiotropic effects of the MSTN gene

MSTN is widely involved in the regulation of metabolic and skeletal systems under physiological conditions, suggesting that perturbation of its function may have a profound effect. In fact, many studies have shown that MSTN overexpression is closely related to the occurrence and development of various muscle atrophy diseases, such as cancer cachexia and senile sarcopenia, making it an important potential therapeutic target (Ahmad et al., 2018; Lee and McPherron, 1999). For example, in an experimental cancer cachexia model, the MSTN signaling pathway was upregulated before the occurrence of muscle atrophy (Samant et al., 2017; Han and Mitch, 2011). The use of ACVRIIB antagonists to block MSTN signaling could effectively reverse muscle atrophy and prolong survival in model animals, providing an important pathophysiological perspective for understanding the pleiotropic effects induced by the loss of MSTN function caused by gene editing.

### 5.1 Metabolic regulatory mechanisms

MSTN gene editing profoundly affects the metabolism of animals and involves global reprogramming of energy metabolism and nutrient distribution. As mentioned above, edited animals generally show reduced fat deposition and improved feed efficiency. These phenotypes are due to the following core mechanisms.

In terms of energy metabolism, MSTN deficiency can significantly increase protein anabolism by alleviating the inhibition of the Akt/mTOR pathway (Oke et al., 2021). Moreover, edited individuals usually exhibit adaptive changes in their basal metabolic rate, directing more energy toward muscle growth (Zhao et al., 2022). At the molecular level, MSTN reduces glucose uptake by inhibiting insulin-dependent and insulin-independent pathways, whereas MSTN deficiency reverses these effects and improves systemic glucose homeostasis (Urnov et al., 2010).

In terms of lipid metabolism, a recent study revealed that in addition to directly inhibiting PPARγ expression (Deng et al., 2017), MSTN deletion can affect the fatty acid desaturation process through MEF2C/miR-222/SCD5 signaling, thereby finely regulating the quality and quantity of lipid deposition (Ren et al., 2020). This metabolic reprogramming has systemic features and is even reflected in germ cells. For example, the expression of mitochondria-related proteins in MSTN-edited bovine sperm was upregulated, suggesting that the energy metabolism pathway underwent extensive adaptive changes.

### 5.2 Physiological and molecular basis of effects on reproductive properties

The effects of MSTN gene editing on the reproductive performance of animals are complex and species specific. The mechanism involves the direct regulation of reproductive energy allocation and organ function. With respect to energy distribution, a reduction in body fat reserves is a key factor that leads to a delay in laying and a decrease in egg production (Liu et al., 2022). The underlying cause involves the energy homeostasis signal represented by leptin, which has a disordered regulatory effect on the timing and intensity of the activation of the hypothalamic–pituitary–gonadal (HPG) axis.

At the level of organ function, studies have revealed species-specific effects. Regarding mammals, the development of the myometrium in the uterine horn is enhanced in MSTN homozygous mutant pigs, and the expression of α-smooth actin (ACTA2) and calcinin is upregulated, suggesting that adaptive changes may occur in the structure and contractile function of the uterus (Zhang et al., 2019). Proteomic analysis of the sperm of MSTN-edited Chinese cattle revealed that the expression of proteins related to sperm movement was upregulated in bulls, which may explain why fertility was not significantly affected (Zhao et al., 2022).

In terms of reproductive efficiency, there are species differences. Studies on poultry have shown that although the muscle mass of MSTN-edited quails significantly increased, the number of eggs was reduced, and the age at laying onset was delayed (Kim et al., 2020; Moroudi et al., 2025). These findings suggest that MSTN may affect follicular development through an energy distribution mechanism. The impact on the reproduction of mammals is more complicated. Although MSTN-edited cattle exhibit enhanced sperm function (Zhao et al., 2022) and normal mating ability, excessive fetal muscle development often leads to an increased rate of dystocia, which becomes a major breeding challenge (Gim et al., 2022a). These differences highlight the key role of species-specific reproductive physiological characteristics and energy distribution strategies in the effects of MSTN editing.

### 5.3 Health and welfare

The effects of MSTN editing on animal health and welfare need to be understood from the perspective of the interactions among the bones, the immune system, and stress.

In terms of bone development, the increase in bone mineral density (BMD) mentioned above is not only due to mechanical stress (Wolff’s law) caused by the increase in muscle mass but also related to the direct regulation of the bone metabolism pathway by MSTN (Cheng et al., 2024). Studies have shown that MSTN deficiency activates the BMP signaling pathway through the upregulation of GDF11 expression, thereby promoting osteogenic differentiation and inhibiting osteoclast formation (Zhang et al., 2019). This activation of the GDF11/BMP pathway is an important direct mechanism through which MSTN editing affects bone quality.

In terms of immune function, there is close “myokine”-mediated crosstalk between muscles and the immune system. Sriram et al. (2011) have shown that MSTN affects the inflammatory response by regulating the NF-κB signaling pathway and that its editing may change the secretion profile of myokines (such as IL-6), thereby affecting immune homeostasis.

The stress response is the core indicator of animal welfare. MSTN-edited pigs showed heightened stress sensitivity, which is related to the substantial metabolic burden caused by the rapid increase in muscle mass, as well as the possible concomitant increase in the degree of mitochondrial dysfunction and oxidative stress (Li et al., 2020a; Sriram et al., 2011).

## 6 Sustainable development of MSTN gene editing

MSTN gene editing has entered a new era from the stage of phenotypic verification to in-depth mechanistic analysis and risk assessment (Chen et al., 2019). The current consensus shows that the key to realizing its application value for breeding lies in how to precisely regulate its functions to balance the conflicting needs of muscle growth and overall animal health.

### 6.1 Technical bottlenecks and the development of precise regulatory tools

A technical bottleneck has driven the development of editing tools toward high precision and high efficiency. Currently, editing efficiency and delivery methods vary across species and are still major limitations, especially in poultry (Kim et al., 2020; Khalil et al., 2017). The more fundamental challenge is that although complete MSTN knockout can significantly increase the lean meat percentage, it is often accompanied by side effects such as dystocia and bone defects, which reveal the risks that accompany uncontrollable loss of function (Gim et al., 2022a; Li et al., 2020a). Therefore, the focus of technological evolution is shifting from “complete knockout” to “fine regulation”.

Next-generation gene editing tools, especially base editors (BEs) and pre-editors (PEs), provide new ideas for overcoming this bottleneck (Anadon et al., 2004). The primary goal is to achieve precise site-specific modification rather than random gene breakage (Anzalone et al., 2019; Moscoso et al., 2020). For example, a base editor can accurately simulate natural beneficial point mutations found in Piedmontese cattle, which is expected to reduce the negative effects of complete protein deletion while maintaining muscle growth. More powerful PEs allow the introduction of subtle mutations in the regulatory region of the MSTN gene so that its expression level is finely downregulated rather than completely turned off, representing an unprecedented possibility for balancing muscle growth and animal health/reproductive performance (Laible et al., 2015). Recent studies have successfully implemented BEs in avian primordial germ cells, validating the feasibility of this pathway (Wang et al., 2024).

### 6.2 Multitrait collaborative breeding strategy

An in-depth understanding of the biological function of MSTN reveals the necessity of pleiotropic management. Existing evidence indicates that MSTN is one of the network hubs that regulates homeostasis. The editing effect involves not only the inhibition of muscle release but also cross-talk with other pathways (such as GDF11/BMP) (Sartori et al., 2013). For example, MSTN deficiency may affect bone metabolism through the upregulation of GDF11 expression, which explains the bone changes observed in mice (Suh et al., 2020).

Therefore, a successful breeding strategy needs to take a systematic perspective into account. Combining MSTN editing and genome selection to simultaneously improve the genetic background related to reproduction and health during the breeding process is an effective way to balance multiple traits (Hai et al., 2023). Wang et al. (2015) successfully bred pigs with dual-gene editing of MSTN and the disease resistance gene CD163, demonstrating the feasibility of achieving synchronous improvement in production and health traits through multigene coediting. This study represents a breakthrough from single-trait breeding to balanced multitrait breeding.

### 6.3 Prospects of dynamic and inducible editing systems

Regulatable gene editing represents a strategic solution to mitigate the lifelong side effects of constitutive MSTN knockout. Unlike permanent editing, this approach enables precise spatiotemporal control (Huang et al., 2019). Technical strategies—such as tissue-specific promoters or inducible Cas9 systems (e.g., Cre-LoxP or drug-induced control)—could theoretically restrict MSTN inhibition to postnatal growth, thereby avoiding adverse effects on critical physiological stages such as reproduction (Anzalone et al., 2019). For future translation, research must prioritize adapting these systems for livestock by developing safe delivery vectors and optimizing inducers (Langley et al., 2002). Success in this endeavor would mark a transformative advance, allowing the industry to harness productivity benefits while upholding stringent welfare standards.

### 6.4 Ethics, regulation and public acceptance

The introduction of MSTN-edited animals to the market involves not only technology but also complex ethical and social considerations. First, the animal welfare of gene-edited animals is the core ethical concern (Wray-Cahen et al., 2024). The problems that may occur in gene-edited individuals, such as skeletal abnormalities and dystocia, must be prioritized for resolution in the application of this technology (Zhou et al., 2022; Li et al., 2020a). Second, the regulatory policies for gene-edited organisms differ across countries. For example, the United States Department of Agriculture (USDA) has relaxed regulations on some CRISPR-edited crops that do not carry exogenous DNA. In contrast, the European Court of Justice (ECJ), in its landmark 2018 ruling, explicitly subsumes organisms obtained by newer mutagenesis techniques, including gene editing, under the existing stringent GMO directives (Critchley et al., 2018). This divergence creates regulatory uncertainty and poses challenges in the global promotion of this technology (Anadon et al., 2004).

In addition, public acceptance largely determines the fate of the product market. The transparency of scientific communication; full emphasis on the sustainable benefits of the technology, such as reducing feed consumption and the environmental footprint and potentially improving animal health; and early and continuous communication with consumers and stakeholders are the keys to building social trust. Therefore, the formulation of clear ethical guidelines and active promotion of the dialog between scientific and policy circles are indispensable for the sustainable development of MSTN editing and breeding.

### 6.5 Nongenetic auxiliary strategies

In addition to genetic means, the emerging study of the “gut–muscle axis” suggests that the gut microbiome may indirectly affect muscle homeostasis through immune and metabolic pathways (Du et al., 2022). For example, the composition of the gut microbial communities in MSTN-edited sheep changed, which may be related to the observed metabolic advantages (Du et al., 2022; Gao et al., 2013). This study provides new ideas for optimizing the health performance of MSTN-edited animals and alleviating their potential metabolic burden through nongenetic auxiliary means such as nutritional intervention and prebiotic/probiotic supplementation.

## 7 Conclusion

This review was motivated by the critical need to move beyond the narrow focus on the double-muscle phenotype that has dominated the MSTN editing field. Although previous reviews and studies have successfully established its role in muscle hypertrophy, they have largely overlooked the complex pleiotropic network that MSTN governs. We systematically synthesized evidence that MSTN editing exerts profound, species-specific effects on systemic metabolism, reproduction, bone health, and animal welfare, consequences that are imperative for sustainable breeding. Our analysis concludes that the conventional knockout approach, despite its efficacy in enhancing muscling, is inherently limited by these trade-offs. Therefore, the foremost contribution of this review is to champion a paradigm shift toward precision modulation—using next-generation editors such as base editors and inducible systems—to fine-tune MSTN activity rather than ablate it. This strategic pivot, integrated with multitrait genomic selection, is essential to unlock the full potential of MSTN editing for developing resilient, productive, and welfare-compatible livestock, thereby aligning genetic gains with the principles of sustainable agriculture.

## References

[B1] Ahmad K. Lee E. J. Moon J. S. Park S. Y. Choi I. (2018). Multifaceted interweaving between extracellular matrix, insulin resistance, and skeletal muscle. Cells 7 (10), 148. 10.3390/cells7100148 30249008 PMC6211053

[B2] Alariqi M. Ramadan M. Yu L. Hui F. Hussain A. Zhou X. (2025). Enhancing specificity, precision, accessibility, flexibility, and safety to overcome traditional CRISPR/cas editing challenges and shape future innovations. Adv. Sci. (Weinh) 12 (28), e2416331. 10.1002/advs.202416331 40548648 PMC12302564

[B3] An M. Raguram A. Du S. W. Banskota S. Davis J. R. Newby G. A. (2024). Engineered virus-like particles for transient delivery of prime editor ribonucleoprotein complexes *in vivo* . Nat. Biotechnol. 42 (10), 1526–1537. 10.1038/s41587-023-02078-y 38191664 PMC11228131

[B4] Anadon A. Roda L. Martinez-Larranaga M. R. (2004). Regulation of genetically modified organisms (GMOs) in the european union: principles of risk assessment. Vet. Hum. Toxicol. 46 (6), 340–341. 15587259

[B5] Anzalone A. V. Randolph P. B. Davis J. R. Sousa A. A. Koblan L. W. Levy J. M. (2019). Search-and-replace genome editing without double-strand breaks or donor DNA. Nature 576 (7785), 149–157. 10.1038/s41586-019-1711-4 31634902 PMC6907074

[B6] Ayuti S. R. Lamid M. Warsito S. H. Al-Arif M. A. Lokapirnasari W. P. Rosyada Z. N. A. (2024). A review of myostatin gene mutations: enhancing meat production and potential in livestock genetic selection. Open Vet. J. 14 (12), 3189–3202. 10.5455/OVJ.2024.v14.i12.4 39927343 PMC11799654

[B7] Bialek P. Parkington J. Li X. Gavin D. Wallace C. Zhang J. (2014). A myostatin and activin decoy receptor enhances bone formation in mice. Bone 60, 162–171. 10.1016/j.bone.2013.12.002 24333131

[B8] Carnac G. Vernus B. Bonnieu A. (2007). Myostatin in the pathophysiology of skeletal muscle. Curr. Genom 8 (7), 415–422. 10.2174/138920207783591672 19412331 PMC2647158

[B9] Chen K. Wang Y. Zhang R. Zhang H. Gao C. (2019). CRISPR/Cas genome editing and precision plant breeding in agriculture. Annu. Rev. Plant Biol. 70, 667–697. 10.1146/annurev-arplant-050718-100049 30835493

[B10] Chen M. M. Zhao Y. P. Zhao Y. Deng S. L. Yu K. (2021). Regulation of myostatin on the growth and development of skeletal muscle. Front. Cell Dev. Biol. 9, 785712. 10.3389/fcell.2021.785712 35004684 PMC8740192

[B11] Chen M. Lian D. Li Y. Zhao Y. Xu X. Liu Z. (2023). Global long noncoding RNA expression profiling of MSTN and FGF5 double-knockout sheep reveals the key gatekeepers of skeletal muscle development. DNA Cell Biol. 42 (3), 163–175. 10.1089/dna.2022.0574 36917699

[B12] Chen M. M. Zhao Y. Yu K. Xu X. L. Zhang X. S. Zhang J. L. (2024). A MSTN(Del73C) mutation with FGF5 knockout sheep by CRISPR/Cas9 promotes skeletal muscle myofiber hyperplasia. Elife 12, RP86827. 10.7554/eLife.86827 39365728 PMC11452178

[B13] Cheng J. Lee J. Liu Y. Wang Y. Duan M. Zeng Z. (2024). Effects of myostatin gene knockout on white fat browning and related gene expression in type 2 diabetic mice. Adv. Clin. Exp. Med. 33 (6), 609–617. 10.17219/acem/171300 37831473

[B14] Choe H. M. Quan B. H. Paek H. J. Luo Z. B. Gao K. Han S. Z. (2022). Altered fibrinogen level and fibrin clot structure in myostatin homozygous mutant pig. Anim. Genet. 53 (3), 307–316. 10.1111/age.13187 35285059

[B15] Clop A. Marcq F. Takeda H. Pirottin D. Tordoir X. Bibe B. (2006). A mutation creating a potential illegitimate microRNA target site in the myostatin gene affects muscularity in sheep. Nat. Genet. 38 (7), 813–818. 10.1038/ng1810 16751773

[B16] Cong L. Ran F. A. Cox D. Lin S. Barretto R. Habib N. (2013). Multiplex genome engineering using CRISPR/Cas systems. Science 339 (6121), 819–823. 10.1126/science.1231143 23287718 PMC3795411

[B17] Crispo M. Mulet A. P. Tesson L. Barrera N. Cuadro F. dos Santos-Neto P. C. (2015). Efficient generation of Myostatin knock-out sheep using CRISPR/Cas9 technology and microinjection into zygotes. PLoS One 10 (8), e0136690. 10.1371/journal.pone.0136690 26305800 PMC4549068

[B18] Critchley C. Nicol D. Bruce G. Walshe J. Treleaven T. Tuch B. (2018). Predicting public attitudes toward gene editing of germlines: the impact of moral and hereditary concern in human and animal applications. Front. Genet. 9, 704. 10.3389/fgene.2018.00704 30687386 PMC6334182

[B19] Deng B. Zhang F. Wen J. Ye S. Wang L. Yang Y. (2017). The function of myostatin in the regulation of fat mass in mammals. Nutr. Metab. 14, 29. 10.1186/s12986-017-0179-1 28344633 PMC5360019

[B20] Dong Q. Dong L. Zhu Y. Wang X. Yan X. (2025). Epigenetic silencing of MSTN via m6A modification underlies the renoprotective effects of engineered MSC exosomes with RBM15 depletion in diabetic nephropathy. Funct. Integr. Genomics 25 (1), 244. 10.1007/s10142-025-01746-3 41247540

[B21] Du C. Zhou X. Zhang K. Huang S. Wang X. Zhou S. (2022). Inactivation of the MSTN gene expression changes the composition and function of the gut microbiome in sheep. BMC Microbiol. 22 (1), 273. 10.1186/s12866-022-02687-8 36368924 PMC9650872

[B22] Gao F. Kishida T. Ejima A. Gojo S. Mazda O. (2013). Myostatin acts as an autocrine/paracrine negative regulator in myoblast differentiation from human induced pluripotent stem cells. Biochem. Biophys. Res. Commun. 431 (2), 309–314. 10.1016/j.bbrc.2012.12.105 23291166

[B23] Gim G. M. Kwon D. H. Eom K. H. Moon J. Park J. H. Lee W. W. (2022a). Production of MSTN-mutated cattle without exogenous gene integration using CRISPR-Cas9. Biotechnol. J. 17 (7), e2100198. 10.1002/biot.202100198 34247443

[B24] Gim G. M. Uhm K. H. Kwon D. H. Kim M. J. Jung D. J. Kim D. H. (2022b). Germline transmission of MSTN knockout cattle via CRISPR-Cas9. Theriogenology 192, 22–27. 10.1016/j.theriogenology.2022.08.021 36037573

[B25] Gim G. M. Eom K. H. Kwon D. H. Jung D. J. Kim D. H. Yi J. K. (2023). Generation of double knockout cattle via CRISPR-Cas9 ribonucleoprotein (RNP) electroporation. J. Anim. Sci. Biotechnol. 14 (1), 103. 10.1186/s40104-023-00902-8 37543609 PMC10404370

[B26] Gong Y. F. Li X. L. Liu Z. Z. Jin X. M. Zhou R. Y. Li L. H. (2009). SNP detection and haplotype analysis in partial sequence of MSTN gene in sheep. Genetika 45 (12), 1646–1649. 20198976

[B27] Gonzalez-Ponce F. Gamez-Nava J. I. Gomez-Ramirez E. E. Ramirez-Villafana M. Jacobo-Cuevas H. Rodriguez-Jimenez N. A. (2022). Myostatin levels and the risk of Myopenia and rheumatoid Cachexia in women with rheumatoid arthritis. J. Immunol. Res. 2022, 7258152. 10.1155/2022/7258152 35592686 PMC9113862

[B28] Goodman C. A. McNally R. M. Hoffmann F. M. Hornberger T. A. (2013). Smad3 induces atrogin-1, inhibits mTOR and protein synthesis, and promotes muscle atrophy *in vivo* . Mol. Endocrinol. 27 (11), 1946–1957. 10.1210/me.2013-1194 24002653 PMC3805852

[B29] Grobet L. Martin L. J. Poncelet D. Pirottin D. Brouwers B. Riquet J. (1997). A deletion in the bovine myostatin gene causes the double-muscled phenotype in cattle. Nat. Genet. 17 (1), 71–74. 10.1038/ng0997-71 9288100

[B30] Grochowska E. Borys B. Lisiak D. Mroczkowski S. (2019). Genotypic and allelic effects of the myostatin gene (MSTN) on carcass, meat quality, and biometric traits in colored Polish Merino sheep. Meat Sci. 151, 4–17. 10.1016/j.meatsci.2018.12.010 30658164

[B31] Hai C. Bai C. Yang L. Wei Z. Wang H. Ma H. (2023). Effects of different generations and sex on physiological, biochemical, and growth parameters of crossbred beef cattle by *Myostatin* gene-edited Luxi bulls and simmental cows. Anim. (Basel) 13 (20), 3216. 10.3390/ani13203216 37893940 PMC10603717

[B32] Han H. Q. Mitch W. E. (2011). Targeting the myostatin signaling pathway to treat muscle wasting diseases. Curr. Opin. Support Palliat. Care 5 (4), 334–341. 10.1097/SPC.0b013e32834bddf9 22025090 PMC3273421

[B33] Hennebry A. Berry C. Siriett V. O'Callaghan P. Chau L. Watson T. (2009). Myostatin regulates fiber-type composition of skeletal muscle by regulating MEF2 and MyoD gene expression. Am. J. Physiol. Cell Physiol. 296 (3), C525–C534. 10.1152/ajpcell.00259.2007 19129464

[B34] Hickford J. G. Forrest R. H. Zhou H. Fang Q. Han J. Frampton C. M. (2010). Polymorphisms in the ovine myostatin gene (MSTN) and their association with growth and carcass traits in New Zealand Romney sheep. Anim. Genet. 41 (1), 64–72. 10.1111/j.1365-2052.2009.01965.x 19799595

[B35] Hill E. W. McGivney B. A. Gu J. Whiston R. Machugh D. E. (2010). A genome-wide SNP-association study confirms a sequence variant (g.66493737C>T) in the equine myostatin (MSTN) gene as the most powerful predictor of optimum racing distance for Thoroughbred racehorses. BMC Genomics 11, 552. 10.1186/1471-2164-11-552 20932346 PMC3091701

[B36] Huang Y. Ding Y. Liu Y. Zhou S. Ding Q. Yan H. (2019). Optimisation of the clustered regularly interspaced short palindromic repeats (CRISPR)/Cas9: single-guide RNA (sgRNA) delivery system in a goat model. Reprod. Fertil. Dev. 31 (9), 1533–1537. 10.1071/RD18485 31079595

[B37] Joulia D. Bernardi H. Garandel V. Rabenoelina F. Vernus B. Cabello G. (2003). Mechanisms involved in the inhibition of myoblast proliferation and differentiation by myostatin. Exp. Cell Res. 286 (2), 263–275. 10.1016/s0014-4827(03)00074-0 12749855

[B38] Kalds P. Zhou S. Huang S. Gao Y. Wang X. Chen Y. (2023). When less is more: targeting the myostatin gene in livestock for augmenting meat production. J. Agric. Food Chem. 71 (10), 4216–4227. 10.1021/acs.jafc.2c08583 36862946

[B39] Kambadur R. Sharma M. Smith T. P. Bass J. J. (1997). Mutations in myostatin (GDF8) in double-muscled Belgian Blue and piedmontese cattle. Genome Res. 7 (9), 910–916. 10.1101/gr.7.9.910 9314496

[B40] Kerr T. Roalson E. H. Rodgers B. D. (2005). Phylogenetic analysis of the myostatin gene sub-family and the differential expression of a novel member in zebrafish. Evol. Dev. 7 (5), 390–400. 10.1111/j.1525-142X.2005.05044.x 16174033

[B41] Khalil K. Elayat M. Khalifa E. Daghash S. Elaswad A. Miller M. (2017). Generation of myostatin gene-edited channel catfish (*Ictalurus punctatus*) via zygote injection of CRISPR/Cas9 system. Sci. Rep. 7 (1), 7301. 10.1038/s41598-017-07223-7 28779173 PMC5544710

[B42] Kim G. D. Lee J. H. Song S. Kim S. W. Han J. S. Shin S. P. (2020). Generation of myostatin-knockout chickens mediated by D10A-Cas9 nickase. FASEB J. 34 (4), 5688–5696. 10.1096/fj.201903035R 32100378

[B43] Kim D. H. Lee B. Lee J. Bohrer B. M. Choi Y. M. Lee K. (2023). Effects of a myostatin mutation in Japanese quail (*Coturnix japonica*) on the physicochemical and histochemical characteristics of the *pectoralis major* muscle. Front. Physiol. 14, 1172884. 10.3389/fphys.2023.1172884 37064889 PMC10097996

[B44] Kong H. Liu X. Xia K. Guan Y. Zhang J. Zhang Y. (2025). Magnetically driven high-speed rolling nanoclusters for enhanced CRISPR/Cas9 genome editing. ACS Appl. Mater Interfaces 17 (45), 61707–61717. 10.1021/acsami.5c15498 41185939

[B45] Laible G. Wei J. Wagner S. (2015). Improving livestock for agriculture - technological progress from random transgenesis to precision genome editing heralds a new era. Biotechnol. J. 10 (1), 109–120. 10.1002/biot.201400193 25515661

[B46] Langley B. Thomas M. Bishop A. Sharma M. Gilmour S. Kambadur R. (2002). Myostatin inhibits myoblast differentiation by down-regulating MyoD expression. J. Biol. Chem. 277 (51), 49831–49840. 10.1074/jbc.M204291200 12244043

[B47] Lee S. J. McPherron A. C. (1999). Myostatin and the control of skeletal muscle mass. Curr. Opin. Genet. Dev. 9 (5), 604–607. 10.1016/s0959-437x(99)00004-0 10508689

[B48] Lee J. Kim D. H. Lee K. (2020). Muscle hyperplasia in Japanese quail by single amino acid deletion in MSTN propeptide. Int. J. Mol. Sci. 21 (4), 1504. 10.3390/ijms21041504 32098368 PMC7073117

[B49] Lee E. J. Ahmad S. S. Lim J. H. Ahmad K. Shaikh S. Lee Y. S. (2021a). Interaction of fibromodulin and myostatin to regulate skeletal muscle aging: an opposite regulation in muscle aging, diabetes, and intracellular lipid accumulation. Cells 10 (8), 2083. 10.3390/cells10082083 34440852 PMC8393414

[B50] Lee J. McCurdy C. Chae C. Hwang J. Karolak M. C. Kim D. H. (2021b). Myostatin mutation in Japanese quail increased egg size but reduced eggshell thickness and strength. Anim. (Basel) 12 (1), 47. 10.3390/ani12010047 35011151 PMC8749606

[B51] Lee J. Kim D. H. Brower A. M. Schlachter I. Lee K. (2021c). Research note: improved feed efficiency in quail with targeted genome editing in the myostatin gene. Poult. Sci. 100 (8), 101257. 10.1016/j.psj.2021.101257 34174566 PMC8242037

[B52] Lee J. Kim D. H. Brower A. M. Schlachter I. Lee K. (2021d). Effects of myostatin mutation on onset of laying, egg production, fertility, and hatchability. Anim. (Basel) 11 (7), 1935. 10.3390/ani11071935 34209534 PMC8300113

[B53] Lee J. Tompkins Y. Kim D. H. Kim W. K. Lee K. (2022). Increased sizes and improved qualities of tibia bones by myostatin mutation in Japanese quail. Front. Physiol. 13, 1085935. 10.3389/fphys.2022.1085935 36685194 PMC9846741

[B54] Lee J. Kim D. H. Lee K. (2024). Myostatin gene role in regulating traits of poultry species for potential industrial applications. J. Anim. Sci. Biotechnol. 15 (1), 82. 10.1186/s40104-024-01040-5 38825693 PMC11145818

[B55] Li X. M. Zhang M. H. Liu S. M. Feng J. H. Ma D. D. Liu Q. X. (2019). Effects of stocking density on growth performance, growth regulatory factors, and endocrine hormones in broilers under appropriate environments. Poult. Sci. 98 (12), 6611–6617. 10.3382/ps/pez505 31504910 PMC8913966

[B56] Li B. Cui W. Yang J. (2020a). Enhanced skeletal muscle growth in myostatin-deficient transgenic pigs had improved glucose uptake in stretozotocin-induced diabetes. Transgenic Res. 29 (2), 253–261. 10.1007/s11248-020-00194-y 32078127

[B57] Li R. Zeng W. Ma M. Wei Z. Liu H. Liu X. (2020b). Precise editing of myostatin signal peptide by CRISPR/Cas9 increases the muscle mass of Liang Guang small spotted pigs. Transgenic Res. 29 (1), 149–163. 10.1007/s11248-020-00188-w 31927726

[B58] Li X. Zhang M. Feng J. Zhou Y. (2021). Myostatin and related factors are involved in skeletal muscle protein breakdown in growing broilers exposed to constant heat stress. Anim. (Basel) 11 (5), 1467. 10.3390/ani11051467 34065334 PMC8160752

[B59] Liang Y. Chen F. Wang K. Lai L. (2023). Base editors: development and applications in biomedicine. Front. Med. 17 (3), 359–387. 10.1007/s11684-023-1013-y 37434066

[B60] Lin J. Arnold H. B. Della-Fera M. A. Azain M. J. Hartzell D. L. Baile C. A. (2002). Myostatin knockout in mice increases myogenesis and decreases adipogenesis. Biochem. Biophys. Res. Commun. 291 (3), 701–706. 10.1006/bbrc.2002.6500 11855847

[B61] Liu X. H. Bauman W. A. Cardozo C. P. (2018). Myostatin inhibits glucose uptake via suppression of insulin-dependent and -independent signaling pathways in myoblasts. Physiol. Rep. 6 (17), e13837. 10.14814/phy2.13837 30252210 PMC6121119

[B62] Liu J. Pan M. Huang D. Guo Y. Yang M. Zhang W. (2020). Myostatin-1 inhibits cell proliferation by inhibiting the mTOR signal pathway and MRFs, and activating the ubiquitin-proteasomal system in skeletal muscle cells of Japanese flounder *Paralichthys olivaceus* . Cells 9 (11), 2376. 10.3390/cells9112376 33138208 PMC7692286

[B63] Liu X. Y. Choe H. M. Li Z. Y. Jin Z. Y. Chang S. Y. Kang J. D. (2022). Positive growth of smooth muscle in uterine horns of myostatin homozygous mutant gilt. Res. Vet. Sci. 152, 228–235. 10.1016/j.rvsc.2022.07.030 36027840

[B64] Lv Q. Yuan L. Deng J. Chen M. Wang Y. Zeng J. (2016). Efficient generation of myostatin gene mutated rabbit by CRISPR/Cas9. Sci. Rep. 6, 25029. 10.1038/srep25029 27113799 PMC4844959

[B65] Maeta K. Farea M. Nishio H. Matsuo M. (2022). An antisense oligonucleotide against a splicing enhancer sequence within Exon 1 of the MSTN gene inhibits Pre-mRNA maturation to act as a novel Myostatin inhibitor. Int. J. Mol. Sci. 23 (9), 5016. 10.3390/ijms23095016 35563408 PMC9101285

[B66] Mahata B. Biswas K. (2017). Generation of stable knockout mammalian cells by TALEN-mediated locus-specific gene editing. Methods Mol. Biol. 1498, 107–120. 10.1007/978-1-4939-6472-7_7 27709571

[B67] McFarlane C. Vajjala A. Arigela H. Lokireddy S. Ge X. Bonala S. (2014). Negative auto-regulation of myostatin expression is mediated by Smad3 and microRNA-27. PLoS One 9 (1), e87687. 10.1371/journal.pone.0087687 24498167 PMC3909192

[B68] McPherron A. C. Lawler A. M. Lee S. J. (1997). Regulation of skeletal muscle mass in mice by a new TGF-beta superfamily member. Nature 387 (6628), 83–90. 10.1038/387083a0 9139826

[B69] Meloux A. Rochette L. Maza M. Bichat F. Tribouillard L. Cottin Y. (2019). Growth differentiation Factor-8 (GDF8)/Myostatin is a predictor of troponin I peak and a marker of clinical severity after acute myocardial infarction. J. Clin. Med. 9 (1), 116. 10.3390/jcm9010116 31906236 PMC7019567

[B70] Morissette M. R. Cook S. A. Buranasombati C. Rosenberg M. A. Rosenzweig A. (2009). Myostatin inhibits IGF-I-induced myotube hypertrophy through Akt. Am. J. Physiol. Cell Physiol. 297 (5), C1124–C1132. 10.1152/ajpcell.00043.2009 19759331 PMC2777401

[B71] Moroudi R. S. Mahboudi H. Mahboudi F. (2025). The effect of selection on the two important Myostatin gene mutations in the Dareshouri horse in the Middle East. Vet. Med. Sci. 11 (2), e70300. 10.1002/vms3.70300 40104884 PMC12077112

[B72] Moscoso C. G. Potz K. R. Tan S. Jacobson P. A. Berger K. M. Steer C. J. (2020). Precision medicine, agriculture, and genome editing: science and ethics. Ann. N. Y. Acad. Sci. 1465 (1), 59–75. 10.1111/nyas.14266 31721233

[B73] Ni W. Qiao J. Hu S. Zhao X. Regouski M. Yang M. (2014). Efficient gene knockout in goats using CRISPR/Cas9 system. PLoS One 9 (9), e106718. 10.1371/journal.pone.0106718 25188313 PMC4154755

[B74] Oke O. E. Uyanga V. A. Iyasere O. S. Oke F. O. Majekodunmi B. C. Logunleko M. O. (2021). Environmental stress and livestock productivity in hot-humid tropics: alleviation and future perspectives. J. Therm. Biol. 100, 103077. 10.1016/j.jtherbio.2021.103077 34503814

[B75] Ongaro L. Zhou X. Wang Y. Schultz H. Zhou Z. Buddle E. R. S. (2025). Muscle-derived myostatin is a major endocrine driver of follicle-stimulating hormone synthesis. Science 387 (6731), 329–336. 10.1126/science.adi4736 39818879 PMC12199281

[B76] Pagant S. Huston M. W. Moreira L. Gan L. St Martin S. Sproul S. (2021). ZFN-mediated *in vivo* gene editing in hepatocytes leads to supraphysiologic alpha-Gal A activity and effective substrate reduction in Fabry mice. Mol. Ther. 29 (11), 3230–3242. 10.1016/j.ymthe.2021.03.018 33775910 PMC8572137

[B77] Philip B. Lu Z. Gao Y. (2005). Regulation of GDF-8 signaling by the p38 MAPK. Cell Signal 17 (3), 365–375. 10.1016/j.cellsig.2004.08.003 15567067

[B78] Qian L. Tang M. Yang J. Wang Q. Cai C. Jiang S. (2015). Targeted mutations in myostatin by zinc-finger nucleases result in double-muscled phenotype in Meishan pigs. Sci. Rep. 5, 14435. 10.1038/srep14435 26400270 PMC4585837

[B79] Ren H. Xiao W. Qin X. Cai G. Chen H. Hua Z. (2020). Myostatin regulates fatty acid desaturation and fat deposition through MEF2C/miR222/SCD5 cascade in pigs. Commun. Biol. 3 (1), 612. 10.1038/s42003-020-01348-8 33097765 PMC7584575

[B80] Rodgers B. D. Garikipati D. K. (2008). Clinical, agricultural, and evolutionary biology of myostatin: a comparative review. Endocr. Rev. 29 (5), 513–534. 10.1210/er.2008-0003 18591260 PMC2528853

[B81] Samant S. A. Kanwal A. Pillai V. B. Bao R. Gupta M. P. (2017). The histone deacetylase SIRT6 blocks myostatin expression and development of muscle atrophy. Sci. Rep. 7 (1), 11877. 10.1038/s41598-017-10838-5 28928419 PMC5605688

[B82] Sartori R. Schirwis E. Blaauw B. Bortolanza S. Zhao J. Enzo E. (2013). BMP signaling controls muscle mass. Nat. Genet. 45 (11), 1309–1318. 10.1038/ng.2772 24076600

[B83] Sriram S. Subramanian S. Sathiakumar D. Venkatesh R. Salerno M. S. McFarlane C. D. (2011). Modulation of reactive oxygen species in skeletal muscle by myostatin is mediated through NF-kappaB. Aging Cell 10 (6), 931–948. 10.1111/j.1474-9726.2011.00734.x 21771249 PMC5028794

[B84] Suh J. Kim N. K. Lee S. H. Eom J. H. Lee Y. Park J. C. (2020). GDF11 promotes osteogenesis as opposed to MSTN, and follistatin, a MSTN/GDF11 inhibitor, increases muscle mass but weakens bone. Proc. Natl. Acad. Sci. U. S. A. 117 (9), 4910–4920. 10.1073/pnas.1916034117 32071240 PMC7060712

[B85] Tachida Y. Manian K. V. Butcher R. Levy J. M. Pendse N. Hennessey E. (2025). Systematic empirical evaluation of individual base editing targets: validating therapeutic targets in USH2A and comparison of methods. Mol. Ther. 33 (4), 1466–1484. 10.1016/j.ymthe.2025.01.042 39881543 PMC11997516

[B86] Tivesten A. Bollano E. Andersson I. Fitzgerald S. Caidahl K. Sjogren K. (2002). Liver-derived insulin-like growth factor-I is involved in the regulation of blood pressure in mice. Endocrinology 143 (11), 4235–4242. 10.1210/en.2002-220524 12399417

[B87] Urnov F. D. Rebar E. J. Holmes M. C. Zhang H. S. Gregory P. D. (2010). Genome editing with engineered zinc finger nucleases. Nat. Rev. Genet. 11 (9), 636–646. 10.1038/nrg2842 20717154

[B88] Walker R. G. Poggioli T. Katsimpardi L. Buchanan S. M. Oh J. Wattrus S. (2016). Biochemistry and biology of GDF11 and myostatin: similarities, differences, and questions for future investigation. Circ. Res. 118 (7), 1125–1141. 10.1161/CIRCRESAHA.116.308391 27034275 PMC4818972

[B89] Wang K. Ouyang H. Xie Z. Yao C. Guo N. Li M. (2015). Efficient generation of myostatin mutations in pigs using the CRISPR/Cas9 system. Sci. Rep. 5, 16623. 10.1038/srep16623 26564781 PMC4643223

[B90] Wang S. Fang L. Cong L. Chung J. P. W. Li T. C. Chan D. Y. L. (2022a). Myostatin: a multifunctional role in human female reproduction and fertility - a short review. Reprod. Biol. Endocrinol. 20 (1), 96. 10.1186/s12958-022-00969-4 35780124 PMC9250276

[B91] Wang N. Lv L. Huang X. Shi M. Dai Y. Wei Y. (2022b). Gene editing in monogenic autism spectrum disorder: animal models and gene therapies. Front. Mol. Neurosci. 15, 1043018. 10.3389/fnmol.2022.1043018 36590912 PMC9794862

[B92] Wang Z. Chen J. Wen J. Zhang S. Li Y. Wang J. (2024). RNA-seq and ATAC-seq reveal CYP26A1-mediated regulation of retinoic acid-induced meiosis in chicken primordial germ cells. Anim. (Basel) 15 (1), 23. 10.3390/ani15010023 39794966 PMC11718974

[B93] Wang Q. Yang R. Yang N. Wen C. (2025). Can myostatin editing together with gut microbiota modulation produce more and tastier meat? Meat Sci. 231, 109950. 10.1016/j.meatsci.2025.109950 40929825

[B94] Wei R. Yu Z. Ding L. Lu Z. Yao K. Zhang H. (2025). Improved split prime editors enable efficient *in vivo* genome editing. Cell Rep. 44 (1), 115144. 10.1016/j.celrep.2024.115144 39745853

[B95] Wray-Cahen D. Hallerman E. Tizard M. (2024). Global regulatory policies for animal biotechnology: overview, opportunities and challenges. Front. Genome 6, 1467080. 10.3389/fgeed.2024.1467080 39381324 PMC11459211

[B96] Yang M. Liu C. Jiang N. Liu Y. Luo S. Li C. (2023). Myostatin: a potential therapeutic target for metabolic syndrome. Front. Endocrinol. (Lausanne) 14, 1181913. 10.3389/fendo.2023.1181913 37288303 PMC10242177

[B97] Yu S. Yue W. Li S. Chen S. Wu P. (2025). Characteristics identification and mitigating potentials of provincial gaseous reactive nitrogen emissions from livestock and poultry breeding systems in China. J. Environ. Manage 374, 124126. 10.1016/j.jenvman.2025.124126 39826361

[B98] Zhang Y. R. Zhang L. S. Wang Z. Liu Y. Li F. H. Yuan J. M. (2018). Effects of stocking density on growth performance, meat quality and tibia development of Pekin ducks. Anim. Sci. J. 89 (6), 925–930. 10.1111/asj.12997 29682864

[B99] Zhang T. Lu Y. Song S. Lu R. Zhou M. He Z. (2019). Double-muscling’ and pelvic tilt phenomena in rabbits with the cystine-knot motif deficiency of myostatin on exon 3. Biosci. Rep. 39 (5), BSR20190207. 10.1042/BSR20190207 31072915 PMC6527932

[B100] Zhao Y. Yang L. Su G. Wei Z. Liu X. Song L. (2022). Growth traits and sperm proteomics analyses of myostatin gene-edited Chinese yellow cattle. Life (Basel) 12 (5), 627. 10.3390/life12050627 35629295 PMC9147296

[B101] Zhou S. Kalds P. Luo Q. Sun K. Zhao X. Gao Y. (2022). Optimized Cas9: sgRNA delivery efficiently generates biallelic MSTN knockout sheep without affecting meat quality. BMC Genom. 23 (1), 348. 10.1186/s12864-022-08594-6 35524183 PMC9078021

